# Unraveling the Predictors for Delirium and ICU Stay Duration in Patients with Heart Failure and Reduced Ejection Fraction (HFrEF) Undergoing Coronary Artery Bypass Grafting—A Multicentric Analysis

**DOI:** 10.3390/biomedicines12040749

**Published:** 2024-03-28

**Authors:** Christian Jörg Rustenbach, Stefan Reichert, Rafal Berger, Julia Schano, Attila Nemeth, Helene Haeberle, Christophe Charotte, Tulio Caldonazo, Ibrahim Saqer, Shekhar Saha, Philipp Schnackenburg, Ilija Djordjevic, Ihor Krasivskyi, Stefanie Wendt, Lina Maria Serna-Higuita, Torsten Doenst, Christian Hagl, Thorsten Wahlers, Christian Schlensak, Rodrigo Sandoval Boburg

**Affiliations:** 1 Department of Thoracic and Cardiovascular Surgery, German Cardiac Competence Center, Eberhard-Karls-University of Tuebingen, Hoppe-Seyler-Strasse 3, 72076 Tuebingen, Germany; stefan.reichert@med.uni-tuebingen.de (S.R.); julia.schano@med.uni-tuebingen.de (J.S.); attila.nemeth@med.uni-tuebingen.de (A.N.); christian.schlensak@med.uni-tuebingen.de (C.S.); rodrigo.sandoval-boburg@med.uni-tuebingen.de (R.S.B.); 2 Department of Anesthesiology and Intensive Care Medicine, Eberhard-Karls-University of Tuebingen, 72076 Tuebingen, Germany; helene.haeberle@med.uni-tuebingen.de (H.H.); christophe.charotte@med.uni-tuebingen.de (C.C.); 3 Department of Cardiothoracic Surgery, University Hospital of Jena, Friedrich-Schiller-University, 07747 Jena, Germany; tulio.caldonazo@med.uni-jena.de (T.C.); ibrahim.saqer@med.uni-jena.de (I.S.); torsten.doenst@med.uni-jena.de (T.D.); 4 Department of Cardiac Surgery, Ludwig-Maximilians-University, 80539 Munich, Germanyphilipp.schnackenburg@med.uni-muenchen.de (P.S.); christian.hagl@med.uni-muenchen.de (C.H.); 5 German Centre for Cardiovascular Research (DZHK), Partner Site Munich Heart Alliance, 81377 Munich, Germany; 6 Department of Cardiothoracic Surgery, Heart Center, University of Cologne, 50923 Köln, Germany; ilija.djordjevic@uk-koeln.de (I.D.); ihor.krasivskyi@uk-koeln.de (I.K.); stefanie.wendt@uk-koeln.de (S.W.); thorsten.wahlers@uk-koeln.de (T.W.); 7 Institute for Clinical Epidemiology and Applied Biostatistics, Eberhard-Karls-University of Tuebingen, 72074 Tuebingen, Germany; lina.serna-higuita@med.uni-tuebingen.de

**Keywords:** postoperative delirium, ICU stay, heart failure, CABG, OPCAB, HFrEF

## Abstract

**Objective:** This study assesses predictors for postoperative delirium (POD) and ICU stay durations in HFrEF patients undergoing CABG, focusing on ONCAB versus OPCAB surgical methods. **Summary Background Data:** In cardiac surgery, especially CABG, POD significantly impacts patient recovery and healthcare resource utilization. With varying incidences based on surgical techniques, this study provides an in-depth analysis of POD in the context of HFrEF patients, a group particularly susceptible to this complication. **Methods:** A retrospective analysis of 572 patients who underwent isolated CABG surgery with a preoperative ejection fraction under 40% was conducted at four German university hospitals. Patients were categorized into ONCAB and OPCAB groups for comparative analysis. **Results:** Age and Euro Score II were significant predictors of POD. The ONCAB group showed higher incidences of re-sternotomy (OR: 3.37), ECLS requirement (OR: 2.29), and AKI (OR: 1.49), whereas OPCAB was associated with a lower incidence of delirium. Statistical analysis indicated a significant difference in ICU stay durations between the two groups, influenced by surgical complexity and postoperative complications. **Conclusions:** This study underscores the importance of surgical technique in determining postoperative outcomes in HFrEF patients undergoing CABG. OPCAB may offer advantages in reducing POD incidence. These findings suggest the need for tailored surgical decisions and comprehensive care strategies to enhance patient recovery and optimize healthcare resources.

## 1. Introduction

In the intricate realm of cardiac surgery, understanding postoperative delirium (POD) and the duration of intensive care unit (ICU) stays is crucial for optimizing patient trajectories, healthcare resource management, and long-term outcomes. POD, a condition marked by acute disturbances in attention, awareness, and cognition, is notably prevalent following coronary artery bypass grafting (CABG) and varies based on a multitude of patient-specific factors and surgical techniques [1,2]. Its significance extends beyond the immediate postoperative phase, with implications for cognitive and functional recovery, underscoring the need for its early identification and management within the critical care framework [3,4].

Patients with heart failure and reduced ejection fraction (HFrEF) are especially vulnerable, often experiencing exacerbated risks of POD and extended ICU stays. Such prolonged care not only signifies higher morbidity and healthcare utilization but also indicates a delayed return to daily life [5,6]. While the correlation between the severity of heart failure and adverse postoperative outcomes is known, the literature lacks specific insights into the multifactorial nature of these risks in the HFrEF population undergoing CABG [7]. This gap is particularly pronounced in a comparative analysis of different surgical techniques, namely on-pump CABG with cardiopulmonary bypass (ONCAB) versus off-pump coronary artery bypass (OPCAB).

Central to this discussion is the choice of surgical technique: on-pump CABG with cardiopulmonary bypass (ONCAB) versus off-pump coronary artery bypass (OPCAB). ONCAB is traditionally linked to increased systemic inflammatory responses and organ hypoperfusion, potentially heightening the risk of POD and extended ICU stays [3,8]. In contrast, OPCAB, by circumventing cardiopulmonary bypass, is thought to mitigate these risks [9]. However, the relative benefits and safety of OPCAB, especially in the HFrEF cohort, demand further critical evaluation and empirical substantiation. Existing studies have often provided inconclusive or conflicting results, highlighting the need for more targeted research in this area [8,10].

This study aimed to present a comprehensive analysis to elucidate the incidence, predictors, and outcomes of POD and ICU stay duration following both ONCAB and OPCAB in HFrEF patients. By focusing on this specific patient demographic, this study aimed to fill a notable void in current cardiac surgery research. This research is particularly timely and relevant, considering the evolving landscape of cardiac surgery and the increasing prevalence of heart failure in an aging population [11]. The insights gleaned from this study are poised to significantly enhance critical care practices, offering a more informed, patient-centered approach to managing the postoperative course of this high-risk population. By shedding light on these areas, this study aimed to contribute to the broader quest of advancing patient-centered, evidence-based care in the critical care setting.

## 2. Materials and Methods

### 2.1. Selection and Categorization of Patients

This study, retrospective and multicentric in nature, was conducted at four university hospitals in Germany, namely in Tübingen, Jena, Cologne, and Munich. It included patients who underwent isolated coronary artery bypass graft (CABG) surgery from January 2017 through December 2022, with a specific focus on those with a preoperative ejection fraction (EF) of 40% or lower. Criteria for inclusion encompassed patients who had isolated CABG surgery under various conditions—elective, urgent, or emergency—and were diagnosed with heart failure reduced ejection fraction (HFrEF, EF < 40%) as confirmed via echocardiography. Exclusion criteria were patients presenting with preoperative cardiogenic shock necessitating mechanical cardiovascular support, including an intra-aortic balloon pump (IABP) or veno-arterial extracorporeal life support (ECLS). In total, the study comprised 572 patients, divided into two groups based on the surgical approach: those who underwent surgery with cardiopulmonary bypass (ONCAB) and those without it (OPCAB). The choice of surgical technique, including the decision to switch from OPCAB to ONCAB, the type of bypass grafts used, and the number of distal anastomoses, was determined based on individual center policies and the discretion of the attending surgeons. Furthermore, we conducted a subgroup analysis to investigate whether there were differences between the two groups (OPCAB and ONCAB) with respect to delirium.

### 2.2. Description of Surgical Procedures

A comprehensive analysis and explanation of the surgical methods employed in this study, specifically ONCAB and OPCAB, along with their respective techniques and protocols, have been thoroughly presented in a preceding publication. For a detailed exploration of these methodologies, we direct readers to consult our earlier work [9].

### 2.3. Perioperative Parameters with Delirium and ICU Stay Assessment

We evaluated demographic details like sex, age, weight, and BMI. We then assessed preoperative data, including operative risk factors such as the Euro Score II, peripheral arterial disease, previous strokes, and renal insufficiency. Cardiac-specific risks, including left ventricular ejection fraction (LVEF), history of myocardial infarction (NSTEMI/STEMI), diseased coronary vessel count, left main coronary artery involvement, and prior PCIs, were also examined.

We analyzed the vasopressor and inotropic doses of patients when transferred to the intensive care unit (ICU), length of stay (LOS) at the ICU, and duration of mechanical ventilation (MV) in hour (the total LOS in the hospital), as well as postoperative complications such as bleeding, postoperative myocardial infarction, pneumonia, delirium, acute renal insufficiency, need for hemodialysis, and lastly, in-hospital and 30-day mortality.

Additionally, we incorporated the Confusion Assessment Method for the Intensive Care Unit (CAM-ICU) into our preoperative protocol [12]. It was used postoperatively after extubation and conducted once daily during the ICU stay. This tool is crucial for the early detection of delirium, a frequent and severe complication in cardiac surgery, by evaluating mental status changes, inattention, and altered consciousness. This comprehensive approach allowed for an in-depth preoperative assessment, encompassing both physical and cognitive health aspects, to better inform and tailor surgical and postoperative care strategies [4].

### 2.4. Intraoperative Data

This study conducted a comparative analysis of various intraoperative factors, including the nature of grafts utilized for bypass procedures, the total number of bypasses executed, the length of surgical procedures, any conversions from OPCAB to ONCAB, and the volume of intraoperative transfusions (measured in milliliters). A critical metric assessed was the extent of revascularization, quantitatively determined through the execution of a minimum of three bypasses in cases of tri-vessel coronary disease.

### 2.5. Statistical Methods

The present study employed multiple regression analyses to investigate the predictors of delirium occurrence, mortality, and length of stay in the intensive care unit (ICU) following coronary artery bypass graft (CABG) surgery. The data were analyzed using Statistical Package for the Social Sciences (SPSS, IBM Corp. Released 2021. IBM SPSS Statistics for Windows, Version 28.0. IBM Corp., Armonk, NY, USA) version 28 and R (Version 4.3.2, R Core Team 2023).

Categorical variables were presented as total and relative frequencies. Normally distributed, continuous variables were presented as means and standard deviations (SDs). Non-normally distributed variables were presented as medians and interquartile ranges. Categorical baseline characteristics were compared based on the OPCAB vs. ONCAB group using a Chi-square test or Fisher’s exact test, as appropriate. Normally distributed continuous variables were analyzed using an independent sample T test. If the continuous variables were non-normally distributed, they were analyzed using the Mann–Whitney U-test. A normal distribution was assessed using a Histogram, Q-Q plots, and skewness.

Serum creatinine levels, GFRs, hemoglobin values, lactate, troponin values, creatinekinase (CK), and CK-MB were measured before and after surgery. The GFR was measured with the Cockroft–Gault equation. Analyses of covariance (ANOVAs) were used to evaluate some laboratory results by adjusting baseline values and the inter-group differences between OPCAB and ONCAB.

To pinpoint significant predictors linked to the event, we implemented univariate binary logistic regression analysis. Following this, multivariable binary logistic regression analysis facilitated the identification of independent risk factors for the event. We employed odds ratios (ORs), along with 95% confidence intervals (CIs) and *p*-values, to quantify the risk associated with each event within the OPCAB and ONCAB cohorts. The model’s ability to predict outcomes was evaluated through the receiver operating characteristic (ROC) curve area. To determine the cut-off value for the investigated variables, the Youden Index was calculated from the ROC curve analysis, identifying the targeted variable with the highest combined sensitivity and specificity. Model comparisons were made using the log-likelihood test (nested models). Model calibration was assessed with the Hosmer–Lemeshow goodness-of-fit test. The best fit model was used as the final model.

#### 2.5.1. Prediction of Delirium Occurrence

To identify predictors of postoperative delirium, a binary outcome logistic regression analysis was performed. Independent variables included a comprehensive range of clinical and demographic factors. The model’s goodness-of-fit was assessed using the Hosmer–Lemeshow test, and the discriminative ability was evaluated using the area under the receiver operating characteristic (ROC) curve.

#### 2.5.2. Duration of ICU Stay

For the analysis of the duration of the ICU stay, two approaches were adopted. First, a linear regression analysis was conducted with the length of ICU stay as the continuous dependent variable. This analysis aimed to identify the impact of various patient and procedural characteristics on the duration of ICU stay.

As an alternative, a survival analysis using the Cox proportional hazards model was utilized to account for censored data due to patients being discharged from the ICU. This method facilitated the examination of the time to event data, with the event being discharge from the ICU. The proportional hazard assumption was verified, and the results were reported as hazard ratios (HRs) with corresponding 95% CIs.

For all analyses, multicollinearity was assessed using variance inflation factors (VIFs), and potential interactions were tested. A two-tailed *p*-value of less than 0.05 was considered to indicate statistical significance.

### 2.6. International Review Board

This project was approved by the IRB of the Tübingen University Hospital with project number 216/2022BO2 from 12 April 2022. Due to the retrospective nature of this study, written consent was not necessary.

## 3. Results

### 3.1. Baseline Characteristics 

Our cohort comprised 572 patients who underwent CABG, categorized into two groups: 474 patients did not develop delirium (no-delirium group), while 98 patients experienced delirium (delirium group). The demographic analysis revealed a predominance of males in the entire cohort (87.8%), with a marginally higher proportion of females in the delirium group (17.3% vs. 11.2% in no-delirium group). A significant age difference was observed between the groups. The average age in the delirium group was higher at 69.7 years, compared to 67.4 years in the no-delirium group (*p* = 0.037). An analysis of baseline health characteristics like BMI, diabetes, hypertension, and renal insufficiency showed similar distributions across both groups, suggesting comparable health profiles at the time of surgery (Table 1 and Table 2).

### 3.2. Intra- and Postoperative Parameters 

In the context of surgical specifics, the median anastomosis count during CABG was uniform across the patient cohorts. A critical observation was the notably higher incidence of complete revascularization in the delirium subgroup (90.6%) in contrast to those without delirium (78.2%, *p* < 0.001). The group with delirium manifested an increased requirement for intraoperative transfusions, encompassing packed red blood cells, platelets, and fresh frozen plasma, with these differences being statistically significant (*p* < 0.05). Postoperatively, the delirium subgroup experienced extended hospitalizations, prolonged ICU stays, and increased durations of mechanical ventilation, with statistical significance (*p* < 0.001). There is a significant variance in delirium rates, with OPCAB at 12.4% and ONCAB at 20.3%, yielding an odds ratio (OR) of 1.53 (95% CI: 0.93–2.52, *p* = 0.019) (Table 3).

### 3.3. Univariate Analysis of Delirium Predictors 

In identifying potential predictors of delirium, age and Euro Score II emerged as significant factors. Each year increase in age was associated with a higher risk of delirium (*p* = 0.038), as was an increase in Euro Score II (*p* = 0.036). Other factors, including sex, BMI, and smoking history, did not demonstrate a statistically significant correlation with the occurrence of delirium post-CABG.

Further analyses were conducted to determine the cut-off values for the occurrence of postoperative delirium (POD) in relation to various variables. In patients who were older than 69.5 years, the risk of postoperative delirium begins to increase significantly (Youden Index, 0.177; sensitivity, 0.622; specificity, 0.555), which is the same as in patients with a Euro Score II higher than 2.650 having an increased risk of postoperative delirium (Youden Index, 0.097; sensitivity, 0.918; specificity, 0.179). Also, the incision to closure time is more than 208 min, and the risk for POD begins to significantly increase (Youden Index, J; sensitivity, 0.571; specificity, 0.609) (Table 4).

### 3.4. Multivariable Logistic Regression Analysis

When considering multiple variables together, several factors stood out as significant predictors of delirium in the entire patient cohort; these included age, Euro Score II, occurrence of STEMI, presence of carotid stenosis, longer incision to closure time, requirement of mechanical ventilation, lower preoperative LVEF, and history of previous PCI discribed in Table 4. We performed a subanalysis according to the operative strategy (ONCAB versus ONCAB). Different predictors were significant for the ONCAB and OPCAB groups. In the ONCAB group, Euro Score II, carotid stenosis, and previous PCI were significant predictors. Conversely, in the OPCAB group, age and preoperative LVEF were identified as significant factors influencing the occurrence of delirium (see Table 5). The binary logistic regression analysis of postoperative complications (Table 6) reveals, the incidence of re-sternotomy was 8.1% in the delirium group compared to 5.9% in the non-delirium group, indicating a higher risk, although not statistically significant (OR = 3.37, *p* = 0.361). ECLS was required by 14.3% of patients with delirium compared to 6.8% without delirium, suggesting a considerably increased risk (OR = 2.29, *p* = 0.013). AKI occurred in 25.5% of patients with delirium versus 10.3% without delirium, indicating a significantly increased risk (OR = 1.49, *p* < 0.001). The need for dialysis was present in 20.4% of patients with delirium compared to 9.7% without delirium, also showing a significantly increased risk (OR = 2.99, *p* = 0.002). Patients undergoing OPCAB had a lower incidence of delirium (29.6%) compared to those undergoing On-Pump Coronary Artery Bypass (ONCAB) (70.4%), suggesting a possible advantage of this technique (OR = 0.68 for OPCAB, *p* < 0.001). The rate of stroke was significantly higher in patients with delirium at 10.2% compared to 1.9% without delirium (OR = 4.69, *p* < 0.001). Sepsis occurred in 24.5% of patients with delirium versus 3.4% without delirium, indicating a substantially increased risk (OR = 4.47, *p* < 0.001). The need for resuscitation was not significantly different in patients with delirium compared to those without (OR = 0.35, *p* = 0.255) as same as the 30-day mortality rate was not significantly different at 4.1% in patients with delirium and 5.3% in those without delirium (OR = 1.40, *p* = 0.638). These results highlight the importance of monitoring and preventing certain complications, particularly ECLS, AKI, and sepsis, in CABG patients to minimize the risk of postoperative delirium.

### 3.5. Predictors of ICU Stay Duration

In examining the factors influencing the length of ICU stay, our analysis identified several key predictors. The duration of ICU stay was significantly associated with variables such as the complexity of the surgical procedure (such as the number of anastomoses performed and size of targeted coronaries, among others), intraoperative complications, and postoperative recovery parameters. Specifically, patients requiring intraoperative blood transfusions, those with longer operative times, and those experiencing postoperative complications like acute kidney injury (AKI) or the need for mechanical ventilation tended to have longer ICU stays. Notably, these associations were similar across both the ONCAB and OPCAB groups, although the relative impact of each factor varied between the two.

## 4. Discussion

In this retrospective study, we analyzed the pre-, intra-, and postoperative data of 572 patients with HFrEF undergoing CABG surgery. We looked for predictors of delirium and length of ICU stay.

Our study’s findings identify age and Euro Score II as significant predictors of delirium in patients with heart failure and reduced ejection fraction (HFrEF) undergoing coronary artery bypass grafting (CABG), which aligns with prior research [13]. Studies have consistently shown that older age and higher preoperative risk scores are associated with an increased likelihood of postoperative complications [14]. For instance, a retrospective study evaluating risk factors for postoperative delirium in cardiac surgery emphasized the role of age and surgical duration as key contributors to delirium incidence, further noting the link between delirium and an increase in postoperative complications like myocardial infarction and respiratory issues [15]. Interestingly, our observation of a higher rate of complete revascularization in the delirium group could suggest that more extensive surgical interventions may contribute to higher stress levels, potentially leading to delirium. This hypothesis finds support in the literature emphasizing the complexity of surgical procedures as a significant factor in postoperative delirium development. A comprehensive analysis of delirium in the context of cardiac surgery highlighted the influence of procedural complexity on delirium’s duration and severity [16]. Moreover, our findings about the frequent need for intraoperative blood transfusions in the delirium group resonate with the existing literature that associates perioperative blood transfusion with an increased risk of postoperative delirium. This relationship was underscored in a systematic review that discussed the broader implications of delirium following major surgeries, including vascular and cardiac procedures [13].

The distinct predictors identified in the ONCAB and OPCAB subgroups emphasize the importance of surgical technique in patient outcomes. The findings suggest that different factors may predispose patients to delirium based on the type of surgical procedure they undergo. For ONCAB, the procedural factors like Euro Score II and previous PCIs were more predictive, whereas, for OPCAB, patient-specific factors like age and LVEF played a more significant role.

In comparing our observed incidence of delirium at 20%, it is pertinent to note the wide range of delirium incidences reported in the existing literature, which spans from 3% to as high as 52% depending on various factors such as patient demographics, surgical procedures, and assessment methodologies [13]. This variability underscores the complexity of accurately predicting POD and highlights the necessity of context-specific evaluations. Studies reporting a 21%, and respectively 39%, incidence [17] and systematic reviews identifying an incidence range of 3% to 52% [13,18] provide a pertinent benchmark for our findings. In particular, our study’s alignment with these reported ranges reinforces the relevance of surgical technique, alongside age and the Euro Score II, as pivotal considerations in POD risk management.

Our analysis revealed that on-pump coronary artery bypass grafting (ONCAB) is associated with a higher incidence of postoperative delirium compared to off-pump coronary artery bypass grafting (OPCAB). This disparity likely stems from the systemic inflammatory responses and microembolic events induced by cardiopulmonary bypass (CPB) usage in ONCAB procedures. Such inflammatory responses and cerebral microemboli are acknowledged factors contributing to cognitive dysfunction following surgery. By circumventing CPB, OPCAB potentially lessens these risks, thereby offering a protective effect against POD development. These insights affirm the critical need for the careful selection of surgical methods, underscoring how procedural choices can significantly affect postoperative cognitive outcomes.

Understanding these predictors is vital for preoperative risk stratification and planning. Tailoring perioperative care to mitigate identified risk factors, such as the careful management of blood transfusions and meticulous decision making in surgical techniques, could potentially reduce the incidence of delirium. The use of the Confusion Assessment Method for the Intensive Care Unit (CAM-ICU) in our preoperative protocol highlights the importance of early recognition and potential prevention strategies for delirium.

The identification of specific cut-off values for age, Euro Score II, and incision to closure time in predicting the occurrence of postoperative delirium (POD) adds a new dimension to our understanding of delirium risk factors. This pivotal finding regarding the establishment of precise cut-off values for age, Euro Score II, and incision to closure time significantly advances our comprehension of the multifaceted risk landscape for postoperative delirium (POD). By systematically analyzing our patient data, we were able to pinpoint these thresholds, which serve as critical markers for increased POD risk. For instance, the age threshold of 69.5 (Youden Index, 0.177; sensitivity, 0.622; specificity, 0.555) years delineates a significant increase in delirium risk, underscoring the heightened vulnerability of older patients to this complication. Similarly, a Euro Score II exceeding 2.65 (Youden Index, 0.097; sensitivity, 0.918; specificity, 0.179) emerges as a clear indicator of elevated delirium risk, reflecting the integral role of preoperative health status in postoperative cognitive outcomes. Moreover, the threshold for incision to closure time at 208 min (sensitivity, 0.571; specificity, 0.609) reveals the significant impact of surgical duration on POD risk, suggesting that prolonged procedures may exacerbate patient susceptibility to delirium.

These findings not only enhance our theoretical understanding of POD risk factors, but also offer tangible benefits for clinical practice. By integrating these cut-off values into preoperative assessments, healthcare providers can identify patients at high risk for delirium more accurately, enabling the implementation of targeted interventions to mitigate this risk. For example, modifications to surgical planning, enhanced monitoring protocols, and personalized patient care strategies can be devised, based on these identified risk thresholds. Consequently, our study not only contributes to the academic discourse on POD, but also paves the way for improved patient outcomes through the application of evidence-based preventive measures. This study sheds light on the complex interplay between postoperative complications and delirium incidence in patients with heart failure and reduced ejection fraction (HFrEF) undergoing coronary artery bypass grafting (CABG). 

The increase in re-sternotomy due to bleeding in the delirium group (8.1%) compared to the no-delirium group (5.9%) was not statistically significant (*p* = 0.361), suggesting that there is no definitive link between re-sternotomy and delirium. This indicates a potential trend that warrants further investigation, albeit with caution due to possible limitations such as sample size and confounders. A notable association was observed between the need for extracorporeal life support (ECLS) and delirium, with patients requiring ECLS having more than a twofold increase in delirium odds (OR = 2.29, *p* = 0.013). This underscores the importance of rigorous monitoring and possible preemptive strategies in managing these high-risk patients.

Renal complications, specifically acute kidney injury (AKI) and the requirement for dialysis, showed a significant prevalence in the delirium group (*p* < 0.001 for AKI; *p* = 0.002 for dialysis). In recognizing the profound impact of these preoperative conditions, our analysis resonates with findings from pivotal studies demonstrating the substantial influence of AKI on long-term mortality in patients with ST-segment elevation myocardial infarction (STEMI) complicated by cardiogenic shock, particularly those undergoing primary percutaneous coronary intervention (PCI) in high-volume tertiary centers [19]. Similarly, the predictive value of LVEF as a determinant of in-hospital mortality among patients with cardiogenic shock, secondary to STEMI, highlights the necessity of incorporating comprehensive preoperative assessments into clinical practice [20]. These considerations are crucial for enhancing patient stratification and tailoring perioperative management strategies to mitigate the risks associated with heart failure and a reduced ejection fraction (HFrEF) in the CABG patient population. Our study, through its multicentric approach and detailed examination of intra- and postoperative outcomes, aimed to contribute to the broader understanding of these dynamics, underscoring the importance of EF and AKI as key factors in the preoperative evaluation process. These findings emphasize the impact of renal health on delirium risk and the critical need for renal protection strategies in the postoperative period. Surgical techniques also influenced delirium incidence.

Off-pump coronary artery bypass (OPCAB) was associated with a lower incidence of delirium compared to on-pump coronary artery bypass (ONCAB), possibly due to differences in procedural factors like systemic inflammation and microembolic events. Interestingly, stroke and sepsis were identified as significant risk factors for delirium, highlighting the necessity for preventive measures against these complications. However, no significant link was found between delirium and outcomes such as resuscitation or 30-day mortality, suggesting that these outcomes may not be directly influenced by delirium in CABG patients. These insights underscore the necessity for a comprehensive approach in managing patients undergoing CABG, balancing surgical technique considerations with vigilant monitoring for complications like ECLS, AKI, stroke, and sepsis.

In the realm of coronary artery disease, the utility of various scoring systems such as the Intermountain Risk Score (IMRS), Naples Prognosis Score (NPS), Prognostic Nutritional Index (PNI), and systemic immune-inflammation indices has been underscored for their predictive value regarding patient outcomes. These scores, through their comprehensive assessment of patient health status and inflammation levels, offer nuanced insights that can significantly enhance the predictive accuracy for endpoints in patients undergoing coronary artery bypass grafting (CABG). By integrating such multifaceted risk assessment tools, clinicians can achieve a more informed and tailored approach to patient care, potentially improving prognostic predictions and patient management strategies. This underlines the imperative of incorporating a broader spectrum of risk scores in future research and clinical practice to refine the prognostication of long-term mortality and other critical outcomes in this patient population.

The findings of our study on postoperative complications and delirium in patients with heart failure and reduced ejection fraction (HFrEF) who underwent coronary artery bypass grafting (CABG) have significant implications for the intensive care unit (ICU) stay duration. Our analysis highlights complications such ECLS requirement and AKI and dialysis, as well as the incidences of stroke and sepsis, notably associated with delirium, which in turn influence the ICU stay. Patients who develop delirium, particularly those with these identified complications, often require longer ICU stays. This extended duration can be attributed to the increased need for monitoring and management of the delirium itself, as well as for associated complications. Furthermore, the choice of surgical technique, with OPCAB being associated with a lower delirium incidence compared to ONCAB, suggests that surgical decisions may also impact the length of ICU stay. In addition to the direct implications of these medical complications, the presence of delirium in postoperative CABG patients often leads to increased staffing needs, given the intensive care and monitoring required for these patients. This not only strains healthcare resources, but also contributes to increased healthcare costs. A prolonged ICU stay, compounded by the need for additional medical interventions and personnel, underscores the economic impact of managing postoperative delirium and its related complications. Therefore, it is crucial to integrate preventive measures, early detection strategies, and efficient management protocols for delirium and its associated complications in the ICU setting. Addressing these factors effectively can potentially reduce ICU stay durations, alleviate the strain on healthcare resources, and decrease the overall cost burden associated with postoperative care in CABG patients. This approach requires a multidisciplinary effort, combining the expertise of surgeons, intensivists, nurses, and other healthcare professionals to optimize patient outcomes and resource utilization in the ICU.

These findings contribute to a more nuanced understanding of delirium risk and could inform the development of targeted strategies for delirium prevention and management, especially in patients identified as high risk based on these parameters.

## 5. Limitations and Future Directions

This study, while comprehensive, is subject to certain limitations that should be acknowledged. The retrospective design may have introduced inherent biases, such as selection bias and potential unrecorded confounders. These factors limit the generalizability of our findings. Additionally, the multicentric nature of the study, involving various hospitals with potentially varying protocols and patient demographics, may have affected the consistency of the results.

Future research should focus on prospective studies to validate and expand upon these findings. Interventional studies aimed at reducing delirium and ICU stay durations in high-risk patients, as identified through preoperative assessments, would be particularly valuable. Further investigations into the underlying mechanisms driving the differences in outcomes between ONCAB and OPCAB procedures would also enhance our understanding of these surgical techniques. Our study primarily focused on clinical outcomes; however, future research should consider patient-centered outcomes post-CABG surgery. Understanding patients’ perspectives on their recovery, quality of life, and overall satisfaction is crucial. This includes assessing physical and mental well-being, pain management, and long-term recovery experiences. Such insights are essential for a holistic approach to patient care, ensuring that treatments align not just with clinical objectives, but also with patient preferences and life quality post-surgery.

## 6. Conclusions

Our findings reveal that factors such as age, Euro Score II, preoperative LVEF, and surgical complexities significantly influence the likelihood of POD, with varying impacts in ONCAB and OPCAB groups. Notably, the ONCAB group showed a higher incidence of POD, influenced more by procedural factors, whereas patient-specific factors like age and preoperative LVEF were more significant in the OPCAB group. The duration of ICU stay was correlated with intraoperative and postoperative factors, including blood transfusion requirements and complications like AKI and mechanical ventilation needs. Our study provides transformative insights into the risk factors for postoperative delirium (POD) in CABG patients, marking a significant advance in cardiac surgery. By identifying critical cut-off values—age of 69.5 years, Euro Score II of 2.650, and incision to closure time of 208 min—we obtained new, precise tools for preoperative risk assessment. This research emphasizes the need for personalized care, balancing procedural and patient-specific factors. Notably, the ONCAB group is more influenced by procedural aspects, while in the OPCAB group, factors like age and preoperative LVEF are crucial. These findings highlight the importance of integrating clinical, demographic, and surgical factors into decision making, ensuring optimized patient outcomes and recovery. Ultimately, this study champions a patient-centered approach in cardiac surgery, where surgical techniques are tailored to each patient’s unique profile, significantly impacting the management of POD risks and enhancing the overall quality of care.

In conclusion, our retrospective study of 572 patients who underwent cardiac surgery underscores the significant roles of age and Euro Score II in predicting postoperative delirium (POD). Crucially, this study highlights the potential of surgical technique selection, specifically the preference for OPCAB over ONCAB, in mitigating the risk of POD.

**Figure 1:**
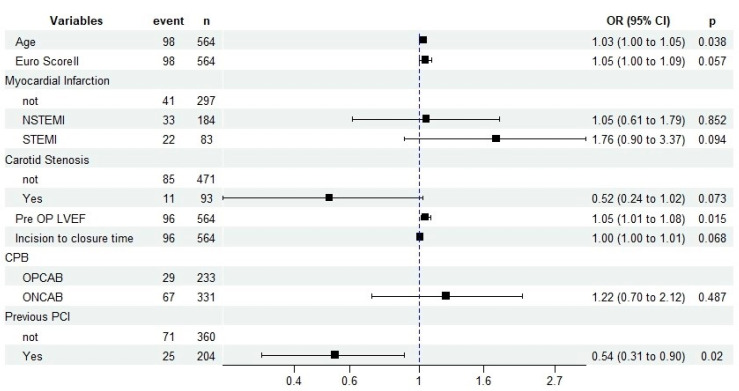
Risk factors for delirium: left decreased risk | right increased risk (forest plot multivariable model).

The key takeaway from our research is the imperative to incorporate these predictive factors into preoperative assessments and surgical planning. By doing so, healthcare professionals can better identify patients at high risk for POD and tailor surgical techniques to minimize this risk, thereby enhancing postoperative recovery outcomes (Figure 1).

## Figures and Tables

**Table 1:** Cohort baseline characteristics.

Variable	Total Cohort (n = 572)	No-Delirium (n = 474)	Delirium (n = 98)	*p* Value
Sex				0.127 ^Chi²^
Male n (%)	502 (87.8%)	421 (88.8%)	81 (82.7%)	
Female n (%)	70 (12.2	53 (11.2%)	17 (17.3%)	
Age mean (±SD)	67.8 (±9.90)	67.4 (±9.94)	69.7 (±9.53)	0.037 ^TT^
BMI mean (±SD)	27.9 (±5.0)	28.0 (±4.83)	27.4 (±5.76)	0.304 ^TT^
Diabetes				0.208 ^Chi²^
OAD n (%)	300 (52.5%)	251 (53.1%)	49 (50.0%)	
Insulin dependent n (%)	134 (23.5%)	115 (24.3%)	19 (19.4%)	
No insulin dependent n (%)	137 (24.0%)	107 (22.6%)	30 (30.6%)	
Smoking history				0.291 ^Chi²^
Not	291 (51.5%)	234 (50.0%)	57 (58.8%)	
Former n (%)	157 (27.8%)	134 (28.6%)	23 (23.7%)	
Active n (%)	117 (20.7%)	100 (21.4%)	17 (17.5%)	
Hypertension yes n (%)	533 (93.2%)	445 (93.9%)	88 (89.8%)	0.215 ^Chi²^
COPD	110 (19.2%)	91 (19.2%)	19 (19.4%)	0.99 ^Chi²^
Hyperlipidemia	483 (84.7%)	400 (84.7%)	83 (84.7%)	0.99 ^Chi²^
Apoplexy, preoperative	59 (10.3%)	49 (10.3%)	10 (10.2%)	0.99 ^Chi²^
Renal insufficiency				0.939 ^Chi²^
Not n (%)	392 (69.5%)	324 (69.5%)	68 (69.4%)	
Grade 1–2 n (%)	104 (18.4%)	85 (18.2%)	19 (19.4%)	
Grade 3–5 n (%)	68 (12.1%)	57 (12.2%)	11 (11.2%)	
Carotid stenosis	94 (16.4%)	83 (17.5%)	11 (11.2%)	0.168 ^Chi²^
Perivascular disease	130 (22.7%)	106 (22.4%)	24 (24.5%)	0.745 ^Chi²^
NYHA Class				0.916 ^Fish^
1 n (%)	16 (3.4%)	4 (4.1%)	20 (3.5%)	
2 n (%)	205 (43.2%)	45 (45.9%)	250 (43.7%)	
3 n (%)	211 (44.5%)	41 (41.8%)	252 (44.1%)	
4 n (%)	42 (8.9%)	8 (8.2%)	50 (8.7%)	
LVEF pre-Op mean (±SD)	31.3 (6.85)	32.5 (7.47)	31.5 (6.97)	0.167 ^TT^
MI and type				0.017 ^Chi²^
Not n (%)	257 (54.3%)	41 (41.8%)	298 (52.2%)	
NSTEMI n (%)	153 (32.3%)	34 (34.7%)	187 (32.7%)	
STEMI n (%)	63 (13.3%)	23 (23.5%)	86 (15.1%)	
Previous PCI	180 (38.1%)	26 (26.5%)	206 (36.1%)	0.041 ^Chi²^
Cardiac decompensation	108 (22.9%)	30 (30.6%)	138 (24.2%)	0.135 ^Chi²^
Type of surgery				0.086 ^Chi²^
Elective n (%)	270 (57.0%)	44 (44.9%)	314 (54.9%)	
Urgent n (%)	137 (28.9%)	35 (35.7%)	172 (30.1%)	
Emergent n (%)	67 (14.1%)	19 (19.4%)	86 (15.0%)	
STS Score median (IQR)	1.90 [0.90, 3.70]	1.80 [0.90, 3.50]	2.20 [1.12, 4.80]	0.014 ^MW^
Euro Score II median (IQR)	3.60 [2.00, 6.93]	3.30 [2.00, 6.38]	4.50 [2.70, 8.50]	0.002 ^MW^

**n** (**%**)**,** Number and percentage of patients in each category; ***p* value**, statistical significance; **Fish**, Fisher’s test; **Chi^2^**, Chi-square test; **TT**, Independent T-test; **MW**, Mann–Whitney U test; **±SD**, Standard Deviation; **OAD**, Oral Antidiabetic Drug; **BMI**, Body Mass Index; **COPD**, Chronic Obstructive Pulmonary Disease; **NYHA Class**, New York Heart Association Classification for grading the severity of heart failure; **LVEF pre-Op**, Left Ventricular Ejection Fraction before surgery; **MI**, Myocardial Infarction; **NSTEMI**, Non-ST Elevation Myocardial Infarction; **STEMI**, ST Elevation Myocardial Infarction; **PCI**, Percutaneous Coronary Intervention; **IQR**, Interquartile Range, showing the middle 50% of data values.

**Table 2:** Univariate analysis of the baseline characteristics related to delirium (n = 572).

Characteristics	n	Event n	OR (95% CI)	*p*-Value
Age	572	98	1.02 (1.00 to 1.05)	0.038
Sex	572	98		
Male			—	
Female			1.67 (0.90 to 2.97)	0.093
BMI	571	98	0.97 (0.93 to 1.02)	0.25
STS Score	571	98	1.08 (0.99 to 1.18)	0.092
Euro Score II	572	98	1.04 (1.00 to 1.08)	0.036
Diabetes mellitus	571	98		
no			—	
OAD			0.85 (0.47 to 1.48)	0.57
Insulin			1.44 (0.86 to 2.38)	0.16
Smoking history	565	97		
non			—	
former			0.70 (0.41 to 1.18)	0.19
active			0.70 (0.38 to 1.24)	0.23
Hypertension	572	98		
yes			0.57 (0.28 to 1.28)	0.15
Hypelipidemia	570	98		
yes			1.0 (0.56–1.88)	0.99
Apoplex	572	98		
yes			0.99 (0.46–1.94)	0.97
Carotid stenosis	572	98		
yes			0.60 (0.29 to 1.12)	0.13
Peripheral vascular disease	572	98		
yes			1.13 (0.67 to 1.85)	0.65
IRC grades	564	98		
non-IRC			—	
IRC grade 1–2			1.07 (0.59 to 1.84)	0.83
IRC grade 3–5			0.92 (0.44 to 1.78)	0.81
LVEF pre-Op	572	98	1.02 (0.99–1.06)	0.14
Clamp time	563	96	1.01 (1.0–1.01)	0.026
Lactate	529	92	0.89 (0.57–1.31)	0.57
Incision to closure	568	98	1 (1.0–1.01)	0.014
Mechanical ventilation	570	96	1 (1.0–1.01)	<0.001

**n**, Total number of patients in the cohort; **Event n**, Number of patients who experienced the event (postoperative delirium); **OR** (**95% CI**), Odds ratio with 95% confidence interval; ***p*-value**, Statistical significance of the association; BMI, Body Mass Index; **STS Score**, Society of Thoracic Surgeons score, predicting mortality and morbidity risk after cardiac surgery; **Euro Score II**, A scoring system used to determine the risk of mortality from cardiac surgery; **OAD**, Oral Antidiabetic Drug; **IRC**, Renal Insufficiency Classification; **LVEF pre-Op**, Left Ventricular Ejection Fraction before operation; **Clamp time,** Duration for which the aortic clamp was applied during surgery; **Lactate**, Blood lactate levels, indicating tissue oxygenation; **Incision to closure**, Total duration from the initial incision to wound closure; **Mechanical ventilation**, Duration for which the patient was mechanically ventilated post-surgery.

**Table 3:** Intra- and postoperative parameters.

Variable		Total Cohort (n = 572)	No-Delirium (n = 474)	Delirium (n = 98)	*p* Value
Number of anastomoses median (IQR)		4 (3–4)	4 (3–4)	4 (4–4)	0.398 ^MW^
Complete revascularization		491 (85.5%)	183 (78.2%)	308 (90.6%)	<0.001 ^Chi²^
**Laboratory values pre-OP**					
Lactate		0.8 [0.6, 1.20]	0.80 [0.60, 1.20]	0.80 [0.6, 1.0]	0.135 ^MW^
Hemoglobin		13.4 (1.95)	13.5 (1.95)	13.2 (1.97)	0.265 ^TT^
GFR		76.4 (25.6)	77.0 (25.6)	73.5 (25.7)	0.215 ^TT^
**Intraoperative Transfusions**					
Packed red blood cells		118 (20.6%)	79 (16.7%)	39 (39.8%)	<0.001 ^Chi²^
Platelets		127 (22.2%)	95 (20.0%)	32 (32.7%)	0.009 ^Chi²^
Fresh frozen plasma		40 (7.0%)	27 (5.7%)	13 (13.3%)	0.014 ^Chi²^
**Intraoperative Vasopressor and Inotropic requirements**					
Epinephrine median (IQR)		0 (0–0.03)	0 (0–0.03)	0 (0–0.04)	0.267 ^MW^
Norepinephrine median (IQR)		0.1 (0.06 –0.16)	0.1 (0.06–0.15)	0.13 (0.06–0.18)	0.07 ^MW^
Dobutamin median (IQR)		0 (0–0)	0 (0–0)	0 (0–0)	0.921 ^MW^
**Postoperative Parameters and Complications**					
Time hospitalization days median (IQR)		13 (9–18)	12 (9–16)	16 (11–23.5)	<0.001 ^MW^
ICU length (hours) median (IQR)		72 (42–144)	70 (37–120)	140 (70–236)	<0.001 ^MW^
Mechanical ventilation (hours) median (IQR)		13 (8–24)	12 (7–21)	18.5 (12–89.5)	0.002 ^MW^

**n**, Number of patients; **IQR**, Interquartile Range; **MW**, Mann–Whitney U test; **Chi^2^**: Chi-square test; **TT,** Independent sample T-test; **GFR**, Glomerular Filtration Rate; **ICU**, Intensive Care Unit (ICU); **[Value] median** (**IQR**)**,** Median value and interquartile range for the data set.

**Table 4:** Risk factors for delirium—entire cohort (n = 572)—(binary logistic regression).

	OR	95% CI		*p* Value	VIF
Age	1.027	1.002	1.053	0.038	1.07
Euro Score II	1.046	0.997	1.094	0.057	1.11
Myocardial infarction					
NSTEMI	1.053	0.611	1.794	0.852	
STEMI	1.756	0.897	3.366	0.094	
Carotid stenosis yes	0.517	0.238	1.023	0.073	1.02
Incision to closure time	1.003	1.000	1.007	0.068	1.13
Mechanical ventilation yes	1.003	1.002	1.005	<0.001	0.08
PreopLVEF	1.046	1.010	1.085	0.015	1.05
ONCAB	1.215	0.705	2.116	0.487	1.11
Previous PCI yes	0.539	0.314	0.897	0.020	1.02

**OR**, Odds Ratio; **95% CI**, 95% Confidence Interval; ***p* value**, Statistical significance; **VIF**, Variance Inflation Factor, assessing multicollinearity in regression analysis; **Euro Score II**, A scoring system used to determine the risk of mortality from cardiac surgery; **NSTEMI**, Non-ST-Elevation Myocardial Infarction; **STEMI**, ST-Elevation Myocardial Infarction; **ONCAB**, Cardiopulmonary Bypass in On-Pump Coronary Artery Bypass surgery; **PCI**, Percutaneous Coronary Intervention; **PreopLVEF**, Preoperative Left Ventricular Ejection Fraction.

**Table 5:** Risk factors for Delirium in ONCAB and OPCAB patients (Binary logistic regression).

ONCAB Surgery				
	OR	95% CI		*p* Value
Age	1.011	0.982	1.042	0.476
Euro Score II	1.082	1.021	1.147	0.007
Myocardial infarction				
NSTEMI	0.984	0.490	1.943	0.963
STEMI	1.345	0.564	3.102	0.493
Carotid stenosis yes	0.303	0.097	0.779	0.022
Incision to closure time	1.004	0.999	1.008	0.095
Mechanical ventilation yes	1.004	1.002	1.006	<0.001
Preop LVEF	1.032	0.990	1.079	0.147
Previous PCI	0.346	0.163	0.686	0.003
**OPCAB Surgery**				
Age	1.070	1.018	1.129	0.010
Euro Score II	0.978	0.878	1.071	0.65
Myocardial infarction				
NSTEMI	0.898	0.331	2.330	0.83
STEMI	1.973	0.585	6.101	0.25
Carotid stenosis yes	0.970	0.292	2.750	0.96
Incision to closure time	1.001	0.993	1.009	0.72
Mechanical ventilation yes	1.002	0.996	1.008	0.34
Preop LVEF	1.074	1.002	1.162	0.060
Previous PCI	0.924	0.391	2.123	0.85

**OR**, Odds Ratio; **95% CI**, 95% Confidence Interval; ***p* value**, Statistical significance; **Euro Score II**, A scoring system used to determine the risk of mortality from cardiac surgery; **NSTEMI**, Non-ST-Elevation Myocardial Infarction; **STEMI**, ST-Elevation Myocardial Infarction; **ONCAB**, Cardiopulmonary Bypass in On-Pump Coronary Artery Bypass surgery; **PCI**, Percutaneous Coronary Intervention; **PreopLVEF**, Preoperative Left Ventricular Ejection Fraction.

**Table 6:** Binary logistic regression of postoperative complications.

Variable	Total Cohort (n = 572)	No-Delirium (n = 474)	Delirium (n = 98)	OR	95% CI	*p* Value
Re-Sternotomy due to bleeding, n (%)	36 (6.4%)	28 (5.9%)	8 (8.1%)	3.37	0.89–12.66	0.361 ^Chi²^
ECLS, n (%)	46 (8.0%)	32 (6.8%)	14 (14.3%)	2.29	1.17–4.48	0.013 ^Chi²^
AKI, n (%)	74 (12.9%)	49 (10.3%)	25 (25.5%)	1.49	0.53–4.17	<0.001 ^Chi²^
Dialysis, n (%)	66 (11.5%)	46 (9.7%)	20 (20.4%)	2.99	0.84–10.56	0.002 ^Chi²^
OPCAB, n (%)	233 (40.7%)	204 (43.0%)	29 (29.6%)	0.68	0.31–1.12	<0.001 ^Chi²^
ONCAB, n (%)	339 (59.3%)	270 (57.0%)	69 (70.4%)	1.79	1.12–2.87	0.014 ^Chi²^
Stroke, n (%)	19 (3.3%)	9 (1.9%)	10 (10.2%)	4.69	1.05–20.86	<0.001 ^Chi²^
Sepsis, n (%)	40 (6.9%)	16 (3.4%)	24 (24.5%)	4.47	1.49–13.97	<0.001 ^Chi²^
Resuscitation, n (%)	37 (6.4%)	14 (2.9%)	5 (5.1%)	0.35	0.06–2.19	0.255 ^Chi²^
30-day mortality, n (%)	29 (5.1%)	25 (5.3%)	4 (4.1%)	1.40	0.65–3.06	0.638 ^Chi²^

**n** (**%**), Number an percentage of patients in each category; **OR**, Odds Ratio; **95% CI**, 95% Confidence Interval; ***p* value**, Statistical significance; **Chi^2^**, Chi-square test; **ECLS**, Extracorporeal Life Support, a medical treatment supporting heart and lung function; **AKI**, Acute Kidney Injury; **OPCAB**, Off-Pump Coronary Artery Bypass, a method of performing bypass surgery without using a heart–lung machine; **ONCAB**, On-Pump Coronary Artery Bypass, a method of surgery where the heart is stopped and blood is pumped through a heart–lung machine; **Re-Sternotomy**—e.g., for bleeding.

## Data Availability

Data are contained within the article, and the foundational research data can be made available upon request in compliance with the EU’s General Data Protection Regulation (GDPR). To ensure compliance, we will seek legal counsel in this matter.
